# Physical activity and the risk of abdominal aortic aneurysm: a systematic review and meta-analysis of prospective studies

**DOI:** 10.1038/s41598-020-76306-9

**Published:** 2020-12-18

**Authors:** Dagfinn Aune, Abhijit Sen, Elsa Kobeissi, Mark Hamer, Teresa Norat, Elio Riboli

**Affiliations:** 1 Department of Epidemiology and Biostatistics, School of Public Health, Imperial College London, St. Mary’s Campus, Norfolk Place, Paddington, London, W2 1PG UK; 2 Department of Nutrition, Bjørknes University College, Oslo, Norway; 3 Department of Endocrinology, Morbid Obesity and Preventive Medicine, Oslo University Hospital, Oslo, Norway; 4 Department of Public Health and Nursing, Faculty of Medicine and Health Sciences, Norwegian University of Science and Technology, Trondheim, Norway; 5 Big Data Institute, University of Oxford, Oxford, UK; 6 Institute Sport Exercise & Health, Division Surgery & Interventional Science, University College London, London, UK

**Keywords:** Epidemiology, Cardiology, Medical research, Risk factors

## Abstract

The association between physical activity and risk of abdominal aortic aneurysm has been inconsistent with some studies reporting a reduced risk while others have found no association. We conducted a systematic review and meta-analysis of prospective studies to quantify the association. PubMed and Embase databases were searched up to 3 October 2020. Prospective studies were included if they reported adjusted relative risk (RR) estimates and 95% confidence intervals (CIs) of abdominal aortic aneurysm associated with physical activity. Summary RRs (95% CIs) were estimated using a random effects model. Nine prospective studies (2073 cases, 409,732 participants) were included. The summary RR for high vs. low physical activity was 0.70 (95% CI: 0.56–0.87, I^2^ = 58%) and per 20 metabolic equivalent task (MET)-hours/week increase of activity was 0.84 (95% CI: 0.74–0.95, I^2^ = 59%, n = 6). Although the test for nonlinearity was not significant (*p* = 0.09) the association appeared to be stronger when increasing the physical activity level from 0 to around 20–25 MET-hours/week than at higher levels. The current meta-analysis suggest that higher physical activity may reduce the risk of abdominal aortic aneurysm, however, further studies are needed to clarify the dose–response relationship between different subtypes and intensities of activity and abdominal aortic aneurysm risk.

## Introduction

Aortic aneurysms are dilatations of the aorta which when ruptured have an 80% overall mortality rate, with about one third dying before reaching hospital^1^ and 25–50% of cases undergoing surgery being fatal^1–4^. Some studies have shown improved survival rates in more recent years^1^. According to the Global Burden of Disease Study aortic aneurysms was estimated to account for 167,200 deaths and 3 million disability-adjusted life years in 2017^5,6^. The vast majority of aortic aneurysms are abdominal aortic aneurysms, with only 3% originating in the thorax^7^. In Caucasian populations the prevalence of abdominal aortic aneurysms is 4.7%^8–10^, while it is 0.45% in Asian populations^11^. Established risk factors for abdominal aortic aneurysm include age^12–14^, male sex^15^, family history of abdominal aortic aneurysm^16^, low education^15^, hypertension^13–15,17^, elevated lipid levels^16^, coronary heart disease^12,15^, peripheral artery disease^12^, chronic obstructive pulmonary disease and smoking^13,15,18^, while diabetes or diabetes medications appears to be protective^12–16,19^.

Physical activity has been shown to reduce blood pressure in randomized controlled trials^20^ and has been associated with reduced risk of hypertension in cohort studies^21–24^, and elevated blood pressure is a strong risk factor for abdominal aortic aneurysms^17^. Although there is strong evidence that physical activity reduces the risk of other vascular disorders such as coronary heart disease^25^, stroke^25^, and heart failure^26^, studies on physical activity and risk of abdominal aortic aneurysm have shown mixed results^13,14,27–33^. Some studies have shown inverse associations between higher physical activity and risk of abdominal aortic aneurysm^27,29–33^, however, other studies found no association^13,14,28^. The equivocal nature of these findings might be explained by relatively low numbers of events in some studies as abdominal aortic aneurysm are far less common than other cardiovascular events such as coronary heart disease and stroke.

Given the high mortality rates among patients with ruptured aortic aneurysm^2^, primary prevention is of major importance to reduce the public health burden of abdominal aortic aneurysms. We therefore conducted a systematic review and meta-analysis of prospective studies on physical activity and the risk of abdominal aortic aneurysm to clarify whether there is an association as well as the strength and shape of the dose–response relationship between the two.

## Results

**Figure 1:**
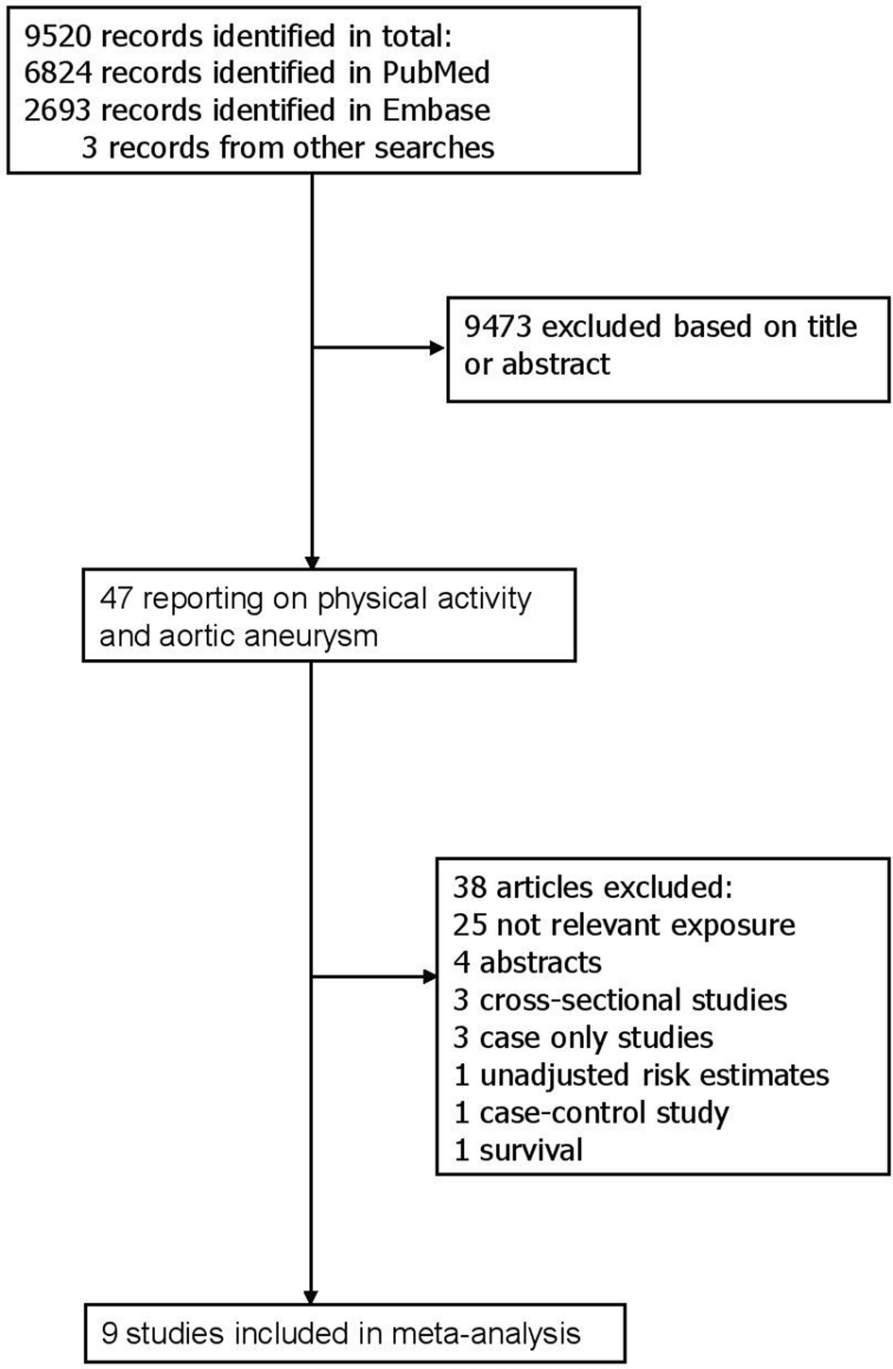
Flow-chart of study selection.

Out of a total of 9520 records retrieved by the search, 9473 were excluded based on title or abstract, and of the 47 publications that were assessed in more detail 9 cohort studies (2073 cases of abdominal aortic aneurysm among 409,732 participants) were included in the meta-analysis (Fig. 1, Table 1)^13,14,27–33^. Five of the studies were from Europe and four studies were from the US. Three studies were identified from searches on other risk factors and abdominal aortic aneurysm^27,28,31^. Eight studies reported on physical activity and abdominal aortic aneurysm^13,14,27,29–33^ and one reported on aortic aneurysm overall^28^, and the latter study was still included in the overall analysis since most aortic aneurysms are abdominal aortic aneurysms (in a sensitivity analysis this study was excluded). Physical activity was assessed by self-reported (most studies) or interviewer-administered (one study) questionnaires, and two studies used validated questionnaires (Table 1). Three studies reported physical activity measures in METs^14,31,32^ two reported the duration of physical activity^30,33^, one reported physical activity on a scale from none to heavy^27^, one used a physical activity index^28^ and two simply compared no activity with any activity^13,29^. For three studies^27,30,33^ the physical activity measures were converted to MET-hours/week as described in the methods section.

**Table 1:** Prospective studies of physical activity and abdominal aortic aneurysm.

References, country	Study name or description	Study period	Number of participants, number of cases	Identification of cases	Physical activity assessment	Type of activity, subgroup	Comparison	Relative risk (95% confidence interval)	Adjustment for confounders
Oyenuga et al.^33^, USA	The Atherosclerosis Risk in Communities (ARIC) study	1987–1989–2011, 22.6 years follow-up	14,375 men and women, age 45–64 years: 545 AAA cases	Self-report confirmed by hospitalization records and death records, linkage to hospital records	Interviewer administered Baecke questionnaire	Leisure-time physical activity	0 min/wk	1.00 0.75 (0.59–0.94) 0.78 (0.64–0.95)	Age, sex, race
							1–149		
							≥ 150		
Hamer et al.^32^, United Kingdom	The Health Survey for England and the Scottish Health Surveys	1994, 1995, 1997, 1998, 1999, 2003, 2004, 2006, 2008–2009/2011, 9.4 years follow-up	65,093 men and women (59,122 without prevalent CVD), age ≥ 40 years: 113 (76) AAA deaths	British National Health Service Central Registry	Validated questionnaire	Leisure-time physical activity—meeting recommendations	Inactive	1.00	Age, sex, smoking, social occupational group, chronic illnesses, psychological distress
							Insufficient	0.51 (0.24–1.11)	
							Sufficient	0.41 (0.10–1.69)	
							High	0.77 (0.24–2.45)	
						Leisure-time physical activity, all	< 1.64 MET-hrs/wk	1.00	
							1.65–9.37	0.91 (0.55–1.52)	
							9.38–19.30	1.29 (0.75–2.20)	
							19.31–37.60	0.85 (0.45–1.61)	
							> 37.60	0.88 (0.43–1.82)	
						Leisure-time physical activity, excluding prevalent CVD at baseline	< 1.64 MET-hrs/wk	1.00	
							1.65–9.37	0.69 (0.36–1.33)	
							9.38–19.30	1.00 (0.52–1.94)	
							19.31–37.60	0.78 (0.37–1.63)	
							> 37.60	0.86 (0.37–1.96)	
Nordkvist et al.^31^, Sweden	Malmö Diet and Cancer Study	1991–1996–NA, 20.7 years follow-up	26,133 men and women, mean age 57.3 years: 353 AAA cases	Linkage to Inpatient and Outpatient Register, Cause of Death Register	Questionnaire	Leisure-time physical activity	0–7.5 MET-hrs/wk	1.00	Age, sex
							7.5–15	0.72 (0.35–1.04)	
							15–25	0.50 (0.35–0.72)	
							25–50	0.54 (0.39–0.74)	
							> 50	0.46 (0.31–0.68)	
Stackelberg et al.^30^, Sweden	Cohort of Swedish Men	1998–2011, 13 years follow-up	14,249 men, age 65–75 years: 156 AAA cases	Ultrasound screening	Validated questionnaire	Walking, bicycling	Almost never	1.00	Age, education, smoking status, pack-years, BMI, waist circumference, healthy diet score, alcohol, diabetes mellitus, cardiovascular disease, hypertension, hypercholesterolemia
							< 20 min/day	0.83 (0.53–1.32)	
							20–40	0.72 (0.45–1.16)	
							≥ 40	0.59 (0.36–0.97)	
Wong et al.^14^, USA	Health Professionals Follow-up Study	1986–2002, ~ 14.6 years follow-up	39,352 men, age 40–75 years: 376 AAA cases	Self-report confirmed by medical records, National Death Index	Questionnaire	Leisure-time physical activity	0.1–5.9 METs/wk	1.00	Age, smoking, hypertension, diabetes, hypercholesterolemia, BMI
							6.0–13.7	0.98 (0.74–1.31)	
							13.8–24.2	1.15 (0.85–1.56)	
							24.3–40.8	0.95 (0.67–1.35)	
							≥ 40.9	1.02 (0.72–1.46)	
Lindblad et al.^29^, Sweden	Malmo Preventive Study	1974–1991, 21 years follow-up	22,444 men, mean age 43.7 years: Nested case–control study: 126 AAA cases 126 controls	Hospital register data, SwedVasc quality control data, death certificates	Questionnaire	Physical inactivity	Yes vs. no	2.67 (1.42–5.01)	Age, serum triglycerides, DBP, serum cholesterol, smoking
Tornwall et al.^13^, Finland	Alpha-Tocopherol, Beta-Carotene Cancer Prevention Study	1985–1988–1993, 5.8 years follow-up	29,133 male smokers, age 50–69 years: 181 AAA cases	National hospital discharge register, national register of causes of death	Questionnaire	Leisure-time exercise	No vs. yes	1.29 (0.95–1.73)	Age, cigarettes per day, years of smoking, BMI, SBP, DBP, total cholesterol, HDL cholesterol, diabetes mellitus, education, exercise, alpha-tocopherol and beta-carotene supplementation group
Goldberg et al.^28^, USA	Honolulu Heart Program	1965–1968–1988, 23 years follow-up	2710 Japanese American men, age 55–64 years: 119 AA cases	Medical, surgical and autopsy records	History of usual 24-h physical activity	Physical activity index	≤ 29.6	1.00	Age, ventricular rate, BMI, SBP, serum cholesterol, serum triglycerides, serum glucose, serum uric acid, hematocrit, forced expiratory volume, cigarettes per day, alcohol
							29.7–32.1	0.46 (0.19–1.13)	
							3.22–35.5	0.97 (0.47–2.01)	
							≥ 35.6	1.37 (0.69–2.72)	
Hammond et al.^27^, USA	Cancer Prevention Study 1	1959–1960–NA, 6 years follow-up	218,435 men, age 50–69 years: 141 AAA deaths	Linkage to death records	Questionnaire	Exercise	Heavy	1.00	Age
							Moderate	1.43 (0.84–2.43)	
							Slight	1.87 (1.13–3.10)	
							None	1.83 (1.12–3.11)	

*AAA* abdominal aortic aneurysm, *AA* aortic aneurysm, *BMI* body mass index, *DBP* diastolic blood pressure, *HDL* high-density lipoprotein, *SBP* systolic blood pressure, *wk* week.

**Figure 2:**
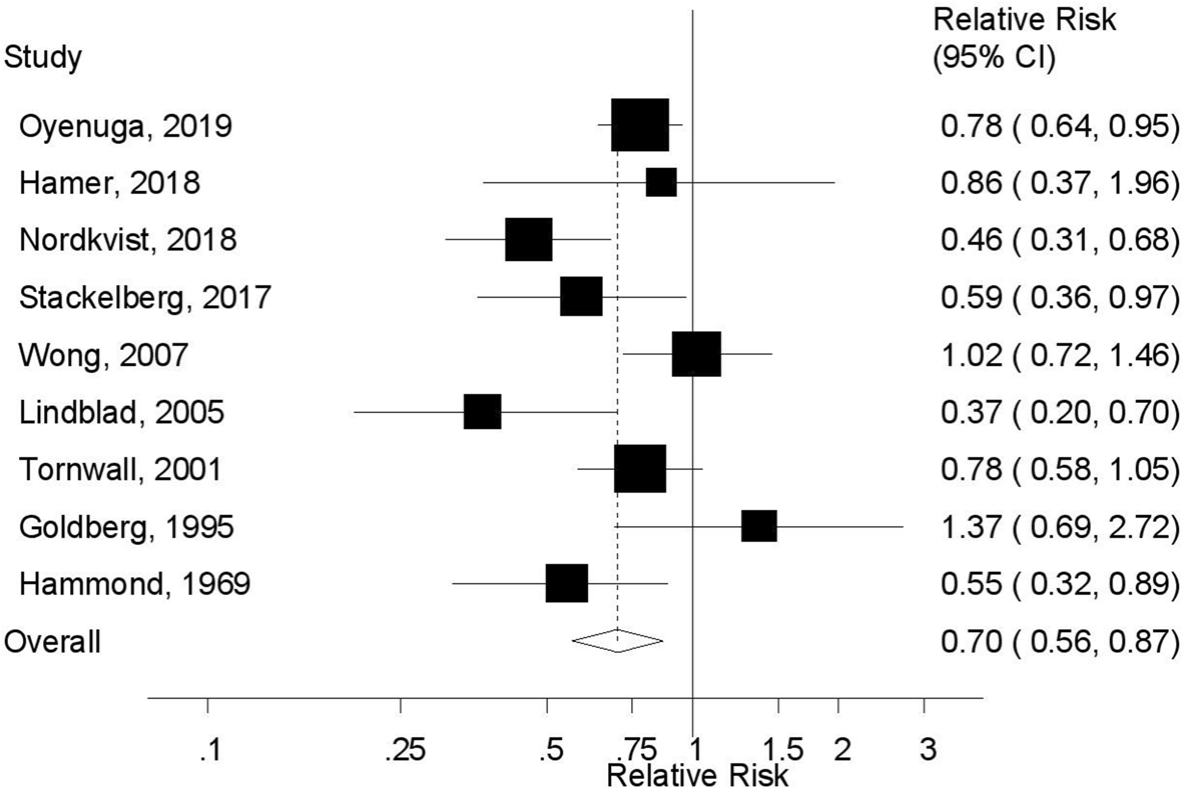
Physical activity and abdominal aortic aneurysm.

The summary RR for individuals with high compared to low physical activity was 0.70 (95% CI: 0.56–0.87, I^2^ = 58%, pheterogeneity = 0.01) (Fig. 2). There was no evidence of publication bias with Egger’s test (*p* = 0.56) or with Begg’s test (*p* = 0.60) or by inspection of the funnel plot (Supplementary Fig. 1). In sensitivity analyses excluding one study at a time the summary RR ranged from 0.66 (95% CI: 0.53–0.83) when excluding the study by Wong et al.^14^ to 0.75 (0.61–0.92) when excluding the study by Nordkvist et al.^31^ (Supplementary Table 2). Excluding one study (Honolulu Heart Program) reporting on total physical activity and aortic aneurysm in Japanese American men^28^ did not materially alter the results, and the summary RR was 0.67 (95% CI: 0.54–0.83, I^2^ = 56%). Further exclusion of one additional study which reported on bicycling/walking^30^ left seven studies on leisure-time physical activity/exercise and aortic aneurysm^13,14,27,29,31–33^ did also not alter the results, and the summary RR was 0.68 (95% CI: 0.54–0.86, I^2^ = 61%).

**Figure 3:**
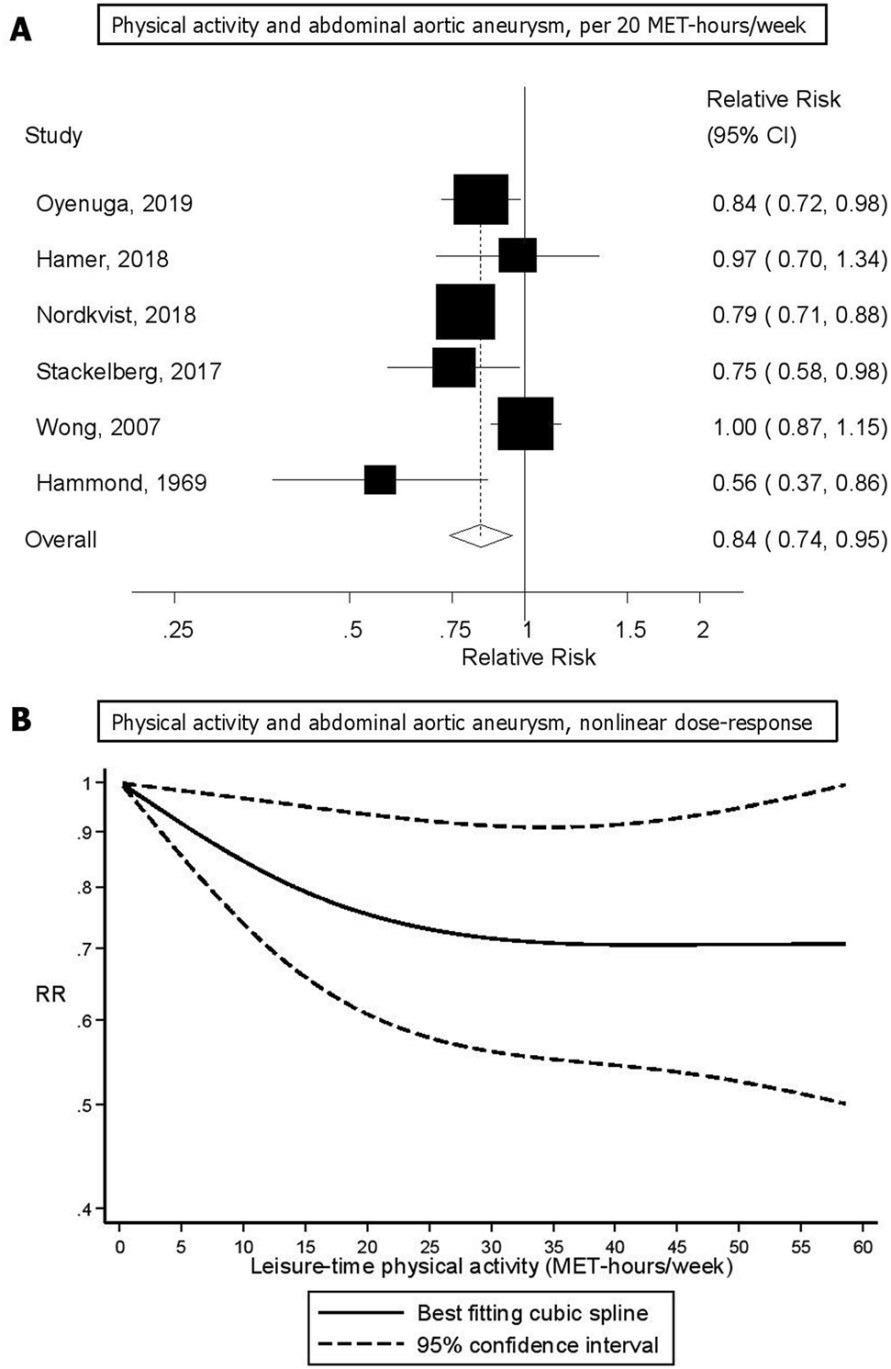
Physical activity and abdominal aortic aneurysm, per 20 MET-hours/week and nonlinear dose–response analysis.

Six studies were included in the dose–response analysis^14,27,30–33^ including 1641 cases and 377,637 participants. The summary RR per 20 MET-hours/week increase in physical activity was 0.84 (95% CI: 0.74–0.95, I^2^ = 59%) (Fig. 3a). There was no indication of publication bias with Egger's test (*p* = 0.71) or with Begg's test (*p* = 0.63). Sensitivity analyses excluding the Cancer Prevention Study 1^27^ (for which we approximated the physical activity level using data from the Cancer Prevention Study 2^34^ did not substantially alter the results and the summary RR was 0.86 (95% CI: 0.77–0.97, I^2^ = 53%) (Supplementary Fig. 3). In influence analyses excluding one study at a time, the summary RR ranged from 0.80 (95% CI: 0.73–0.88) when excluding the study by Wong et al.^14^, to 0.86 (95% CI: 0.77–0.97) when excluding the study by Hammond et al.^27^ (Supplementary Fig. 3). We also repeated the analysis of the highest versus lowest level of activity and risk of abdominal aortic aneurysm using the same studies that were included in the dose–response analysis and the summary RR was 0.69 (95% CI: 0.54–0.88, I^2^ = 55%), which was very similar to the summary estimate among all studies.

Although the test for nonlinearity was not significant (*p* = 0.09) the association appeared to be stronger when increasing the physical activity level from 0 to around 20–25 MET-hours/week than at higher levels (Fig. 3b, Supplementary Table 2).

### Subgroup and sensitivity analyses and study quality

Inverse associations were observed in most subgroup analyses defined by duration of follow-up, outcome type, outcome assessment, number of cases, study quality, and by whether studies excluded prevalent cases at baseline. The associations were often not significant when analyses were stratified by whether the studies adjusted for confounding factors or not, however, the number of studies in some subgroups were limited and there was no significant heterogeneity between any of the subgroup analyses (Table 2).

**Table 2:** Subgroup analyses of physical activity and abdominal aortic aneurysm.

	Physical activity and abdominal aortic aneurysm				
	*n*	Relative risk (95% CI)	*I*^*2*^ (%)	*P*_h_^1^	*P*_h_^2^
All studies	9	0.70 (0.56–0.87)	58.3	0.01	
**Sex**					
Men	6	0.72 (0.53–0.98)	61.9	0.02	0.74
Women	0				
Men and women	3	0.66 (0.44–0.98)	65.3	0.06	
**Outcome type**					
Incidence	7	0.71 (0.55–0.91)	66.3	0.007	0.58
Mortality	2	0.62 (0.40–0.96)	0	0.37	
**Follow-up**					
< 10 years	3	0.73 (0.57–0.93)	0	0.47	0.95
≥ 10 years	6	0.69 (0.51–0.95)	71.7	0.003	
**Geographic location**					
Europe	5	0.58 (0.43–0.79)	48.3	0.10	0.13
America	4	0.85 (0.64–1.12)	51.6	0.10	
**Number of cases**					
Cases < 150	4	0.68 (0.39–1.18)	64.7	0.04	0.81
Cases ≥ 150	5	0.72 (0.57–0.91)	59.9	0.04	
**Exclusion of prevalent cases at baseline**					
Yes	6	0.76 (0.60–0.96)	61.1	0.03	0.24
No	3	0.53 (0.35–0.80)	22.4	0.28	
**Outcome assessment**					
Record linkage (hospital, death records)	8	0.71 (0.56–0.91)	62.0	0.01	0.67
Ultrasound screening	1	0.59 (0.36–0.97)			
**Study quality**					
0–3 stars	0				0.82
4–6 stars	4	0.68 (0.44–1.05)	66.8	0.03	
7–9 stars	5	0.71 (0.54–0.94)	60.0	0.04	
**Adjustment for confounding factors**^**3**^					
Age					
Yes	9	0.70 (0.56–0.87)	58.3	0.01	NC
No	0				
Education					
Yes	2	0.72 (0.56–0.93)	0	0.34	0.97
No	7	0.70 (0.52–0.94)	67.2	0.006	
Alcohol					
Yes	3	0.85 (0.50–1.44)	47.9	0.15	0.43
No	6	0.66 (0.52–0.86)	66.8	0.01	
Smoking					
Yes	5	0.76 (0.54–1.07)	65.1	0.02	0.49
No	4	0.60 (0.45–0.80)	57.6	0.07	
BMI or obesity					
Yes	4	0.86 (0.65–1.14)	43.3	0.15	0.12
No	5	0.58 (0.42–0.80)	60.6	0.04	
Diabetes mellitus					
Yes	3	0.81 (0.61–1.06)	38.7	0.20	0.45
No	6	0.65 (0.47–0.90)	64.9	0.01	
Hypertension					
Yes	2	0.80 (0.47–1.36)	67.8	0.08	0.57
No	7	0.67 (0.52–0.86)	59.4	0.02	
Systolic blood pressure					
Yes	2	0.95 (0.56–1.60)	54.2	0.14	0.23
No	7	0.64 (0.50–0.83)	61.1	0.02	
Diastolic blood pressure					
Yes	2	0.57 (0.27–1.17)	77.5	0.04	0.51
No	7	0.73 (0.57–0.94)	58.3	0.03	
Hypercholesterolemia or serum cholesterol					
Yes	5	0.76 (0.54–1.07)	65.1	0.02	0.49
No	4	0.63 (0.46–0.86)	54.5	0.09	
Triglycerides					
Yes	2	0.71 (0.20–2.55)	86.9	0.006	0.96
No	7	0.71 (0.58–0.87)	47.4	0.08	

*n* denotes the number of studies.

^1^P for heterogeneity within each subgroup.

^2^P for heterogeneity between subgroups with meta-regression analysis.

*BMI* body mass index, *NC* not calculable because no studies were present in one of the subgroups.

The mean (median) study quality scores were 6.8 (7.0) out of 9.0 points possible for the studies included in the analysis. The main reasons for a less than optimal study quality score were lack of representativeness of the general population (n = 3), lack of validation or lack of reporting of validation of physical activity questionnaires (n = 7), lack of exclusion or lack of reporting of exclusion of prevalent AAA cases at baseline (n = 3), and lack of reporting on loss to follow-up (n = 6) (Supplementary Table 3).

## Discussion

This meta-analysis of 9 cohort studies including 2073 cases of abdominal aortic aneurysm among 409,732 participants suggest that a high level of physical activity reduces the risk of abdominal aortic aneurysms by 30%. In the dose–response analysis there was a 16% reduction in the relative risk for each 20 MET-hours/week increase in physical activity (equivalent to approximately 2 h/week of vigorous running or bicycling, 3 h/week of moderate running/bicycling, or 6 h of brisk walking per week) and although the statistical test for nonlinearity was not significant, there was some indication of a stronger reduction in risk from 0 to 20–25 MET-hours/week than at higher levels of activity. The inverse association was observed in European studies, but was not significant in American studies. The association was in the direction of reduced risk, but not always significant across subgroups, most likely because of the limited number of studies in several subgroups. However, there was no evidence of between subgroup heterogeneity with meta-regression analyses.

As with any meta-analysis of epidemiological studies this meta-analysis has limitations. There was moderately high heterogeneity in the analysis across studies. Different studies used different types of questionnaires to assess physical activity, which likely differed in levels of validity that may have contributed to part of the observed heterogeneity. All the included studies assessed physical activity by self-report, and in some studies the self-reported measures were validated. In addition, different studies differed in the way physical activity was reported from a qualitative description of the level of activity to a quantitative measure reported using MET-hours. This appears to be a recurring issue in physical activity epidemiology as we in several previous meta-analyses on physical activity and different health outcomes only were able to include a fraction of the available studies in the dose–response analyses, if at all^35–37^. In the current analysis we were able to include six out of nine studies in the dose–response analysis. It seems less likely that the three studies which were excluded from the dose–response analysis would have substantially altered the overall association as the summary estimate for the highest versus lowest analysis was very similar to the overall analysis when restricted to these same six studies. Any future studies should emphasize reporting the results in a way that can be combined with the available data, for example by reporting the level of physical activity in MET-hours per week and/or hours per week.

We conducted several subgroup analyses to investigate potential sources of heterogeneity, but found no evidence of heterogeneity between subgroups. The association was not significant in among studies with adjustment for BMI or obesity, but whether this is a chance finding, or is an indication that reduced adiposity may be a mechanism that explains part of the association between physical activity and abdominal aortic aneurysm needs further exploration in future studies with models with and without adjustment for adiposity in the same dataset. The non-significant associations among studies with adjustment for hypertension, blood pressure or lipids also needs to be interpreted carefully as these factors potentially could be mediating variables for the association between physical activity and abdominal aortic aneurysm. In addition, we cannot exclude the possibility that the association may have been partly confounded by tobacco smoking, which is a rather strong risk factor for abdominal aortic aneurysm^18^, because the association was not statistically significant when the analyses were restricted to the studies with adjustment for tobacco smoking. However, the association was still in the direction of reduced risk among studies with adjustment for smoking and since there was no significant heterogeneity between these subgroups with meta-regression analyses, limited statistical power may be another potential explanation. Any further studies should adjust more comprehensively for tobacco smoking and analyses stratified by smoking status may be needed to completely rule out the potential for residual confounding.

Reverse causation or a temporal bias where patients with prevalent abdominal aortic aneurysms before baseline may have reduced their physical activity level because of the condition could also have biased the results. However, the association persisted among the six studies that excluded participants with prevalent disease at baseline. Publication bias can affect meta-analyses of published studies. Although we found no evidence of publication bias in this analysis, it is possible that we may have had too low power to detect such bias, however, there was also no evidence of asymmetry in the funnel plots, which might argue against this being the case.

Little is known about the potential underlying mechanisms that could explain a beneficial effect of physical activity on the risk of abdominal aortic aneurysms. Physical activity has been associated with lower blood pressure and a lower risk of hypertension^20–24^, lower lipid levels^38,39^, as well as a lower risk of coronary heart disease^40^, all of which are important risk factors for abdominal aortic aneurysms^12,17,41^. However, it is possible that the association is independent of traditional risk factors. A recent experimental study showed that low intensity exercise improved aortic wall structure and function in a mouse model of Marfan syndrome, while these benefits were attenuated at higher intensity exercise^42^. Reduced expression of matrix metalloproteinases 2 and 9 explained the reduced elastin fragmentation^42^. Whether these findings can be transferred to humans and individuals without Marfan syndrome is unclear and whether other mechanistic pathways are involved needs further study.

The current meta-analysis has several strengths including the relatively large sample size, comprehensive subgroup and sensitivity analyses, detailed linear and nonlinear dose–response analyses and moderately high study quality. Any further studies should investigate the dose–response relationship between different types and intensities of physical activity in relation to risk of abdominal aortic aneurysm, adjust more comprehensively for potential confounding factors, conduct analyses stratified by smoking, and could investigate potential mechanistic pathways that may explain the association between physical activity and lower risk of abdominal aortic aneurysm. In addition, more detailed reporting on the exposure ascertainment and whether it has been validated or not, exclusion of prevalent cases at baseline and/or clear reporting on whether prevalent cases have been excluded at baseline, as well as reporting on loss to follow-up seems to be areas for improving study quality.

The current findings are consistent with recommendations to increase the level of physical activity to reduce the risk of other chronic diseases such as coronary heart disease^25^, stroke^25^, type 2 diabetes^35^, several cancers^43^, and other conditions^36,37^, as well as premature mortality^44^, and suggest these benefits perhaps also may extend to abdominal aortic aneurysms. However, further research is needed before these findings can be considered conclusive.

## Conclusion

In conclusion, this meta-analysis suggests that individuals who are physically active have a 30% reduction in the risk of abdominal aortic aneurysm, however, residual confounding cannot be entirely excluded. Additional studies from geographically diverse regions with better adjustment and stratification for smoking and other potential confounding factors are needed to clarify these associations. Further assessment of the dose–response relationship between different types and intensities of physical activity and risk of abdominal aortic aneurysm and the potential mechanistic pathways that may explain these findings is also needed.

## Material and methods

### Search strategy

Pubmed, and Embase databases were searched up to October 3rd 2020. The full search is described in the Supplementary Text. In addition, we searched the reference lists of the included publications for any additional studies. MOOSE criteria for reporting of meta-analyses were followed^45^.

### Study selection and inclusion criteria

We included published retrospective and prospective cohort studies and nested case–control studies within cohorts which reported adjusted estimates of the relative risk (RR) with the 95% confidence intervals (CIs) for the association between physical activity and the risk of abdominal aortic aneurysm. The excluded studies and exclusion reasons can be found in Supplementary Table 1.

### Data extraction

The following data were extracted from the included studies: The first author’s name, publication year, country where the study was conducted, study period, sample size, number of cases and participants, exposure and subgroup, RRs and 95% CIs and variables adjusted for in the analysis. The data were extracted by DA and checked for accuracy by EK.

### Statistical methods

The random-effects model by DerSimonian and Laird^46^ was used to calculate summary relative risks (RRs) and 95% confidence intervals (CIs) of abdominal aortic aneurysm for the highest vs. lowest level of physical activity and per 20 MET-hours per week. The average of the natural logarithm of the RRs was estimated and the RR from each study was weighted using random effects weights. For the linear dose–response analysis we used the method by Greenland and Longnecker to estimate linear trends and (CIs) across categories of physical activity^47^. For studies that reported physical activity by ranges of activity we calculated the average of the upper and lower cut-off value to obtain an estimate of the midpoint for each category. For studies with open-ended extreme categories we used the width of the adjacent category to estimate an upper and lower cut-off for the highest and lowest category, respectively. Nonlinear dose–response analyses were conducted using restricted cubic splines with knots at 10, 50 and 90% percentiles of the distribution of physical activity, which were combined using multivariate meta-analysis^48,49^.

Because of differences in the way physical activity was reported between studies, conversions to the same scale (MET-hours/week) were made when possible. For one study which reported on walking/bicycling in minutes per day^30^ we used the average of 8 METs for bicycling (equivalent to bicycling at 12–13.9 mph with moderate effort) and 3.3 METs for walking at moderate pace (3.0 mph) ^50^, thus we used 5.65 METs as the average intensity for that study. For a second study we used the average of moderate (3–5.9 METs) and most vigorous (6–12 METs) activities to convert minutes/week of activity to METs (although some vigorous activities, e.g. bicycle racing, competitive skating, and ski racing, have METs up to 18 we chose 12 as an upper limit as most of the vigorous activities are in that range and because relatively few participants would be competitive sportsmen^50^). Thus we used 6.725 METs as intensity for that study. Another study which used a physical activity index^28^ which had a different scoring of the intensities than the MET values was excluded from the dose–response analysis because it was impossible to convert the reported data to METs. For one study (Cancer Prevention Study 1) which reported physical activity data as none, slight, moderate and heavy we used estimated METs from the Cancer Prevention Study 2^34^, but excluded the study in a sensitivity analysis. For one study which reported on aneurysms and peripheral vascular disease combined^32^, the analyses were repeated but restricted to abdominal aortic aneurysm. Two additional studies were only included in the analysis of the highest versus lowest level of physical activity because they had < 3 categories of physical activity^13,29^. For two studies^13,29^ which compared no physical activity with activity we converted the risk estimates so the comparison became the highest vs. the lowest (consistent with the remaining studies) by inverting the risk estimates and confidence intervals.

Heterogeneity between studies was evaluated using Q and I^2^ statistics^51^. I^2^ measures how much of the heterogeneity is due to between study variation rather than chance. We conducted main analyses (all studies combined) and subgroup analyses stratified by study characteristics to investigate potential sources of heterogeneity. The subgroups included sex, outcome type (incidence vs. mortality), duration of follow-up, geographic location, number of cases, exclusion of prevalent cases at baseline, outcome assessment, study quality and by adjustment for confounding factors (age, education, alcohol, smoking, BMI/obesity, diabetes mellitus, hypertension, systolic blood pressure, diastolic blood pressure, hypercholesterolemia or serum cholesterol, and triglycerides. Study quality was assessed using the Newcastle Ottawa scale which rates studies a score from 0 to 9 based on the selection, comparability and outcome assessment of the studies^52^.

Publication bias was assessed using Egger’s test^53^ and Begg-Mazumdar’s test^54^ and by inspection of funnel plots. The statistical analyses were conducted using the software package Stata, version 13.1 software (StataCorp, Texas, US).

## Supplementary information

Supplementary Information.
