# Supplementary material for: Physical activity and the risk of abdominal aortic aneurysm: a systematic review and meta-analysis of prospective studies
Source: Sci Rep. 2020 Dec 18;10:22287. doi: 10.1038/s41598-020-76306-9 (PMC7749100; doi:10.1038/s41598-020-76306-9)
Supplement: Supplementary file 1 — Supplementary Information. [file 41598_2020_76306_MOESM1_ESM.doc]

Supplementary text

PubMed search strategy

1) "physical activity" OR physical activity[MeSH] OR exercise OR exercise[MeSH] OR sports OR sports[MeSH] OR walking OR walking[MeSH] OR biking OR bicycling OR bicycling[MeSH] OR running OR running[MeSH] OR fitness OR "exercise test" OR exercise test[MeSH] OR inactivity OR sedentary OR "risk factor" OR risk factor[MeSH] OR "risk factors" OR risk factors[MeSH]

2) "aortic aneurysm" OR aortic aneurysm[MeSH]

3) "case-control" OR cohort OR prospective OR longitudinal OR retrospective OR "follow-up" OR "cross-sectional" OR "hazard ratio" OR "hazard ratios" OR "relative risk" OR "relative risks" OR "incidence rate ratio" OR "incidence rate ratios" OR "odds ratio" OR odds ratios OR incidence

4) 1 AND 2 AND 3

Embase search strategy

1) physical activity/ or exercise/ or sports/ or walking/ or biking/ or bicycling/ or running/ or fitness/ or exercise test/ or inactivity/ or sedentary/ or risk factor/ or risk factors/

2) (physical activity or exercise or sports or walking or biking or bicycling or running or fitness or exercise test or inactivity or sedentary or risk factor or risk factors).ab,ti.

3) aortic aneurysm.ab,ti.

4) aortic aneurysm/

5) (case-control or cohort or prospective or longitudinal or retrospective or follow-up or cross-sectional or hazard ratio or hazard ratios or relative risk or relative risks or incidence rate ratio or incidence rate ratios or odds ratio or odds ratios or incidence).af.

6) 1 or 2

7) 3 or 4

8) 5 and 6 and 7

Supplementary Table 1. List of excluded studies and exclusion reason

| Exclusion reason | Reference number |
| --- | --- |
| Abstract | (1-4) |
| Case-control study | (5) |
| Case only study | (6-8) |
| Cross-sectional study | (9-11) |
| Not relevant exposure | (12-36) |
| Survival | (37) |
| Unadjusted risk estimates | (38) |

Reference List

(1) Newman AB, Dodson JA, Church T, Bonds D, Buford T, Fielding R, Kritchevsky S, Beavers DP, Pahor M, Stafford RS, Szady AD, McDermott MM. Cardiovascular events in a physical activity intervention as compared to a successful aging intervention: The life study randomized trial. Circulation Conference: American Heart Association's 2014;25.

(2) Buford TW, Cochrane S, Fitzgerald J, Chen S-H, Dodson J, Fielding R, King A, Manini T, Marsh A, McDermott M, Newman A, Pahor M, et al. Objectively-measured physical activity and incident cardiovascular events among mobility limited older adults: The life study. Circulation Conference: American Heart Association's Epidemiology and Prevention/Lifestyle and Cardiometabolic Health 2016.

(3) Singh R, Bodar V, Chen J, Wang L, Sesso HD, Gaziano JM, Djousse L. Healthy lifestyle factors and risk of abdominal aortic aneurysm in the physicians' health study. Cardiology (Switzerland) Conference: 23rd World Congress on Heart Disease International Academy of Cardiology Annual Scientific Sessions 2018;(Supplement 1):2018.

(4) Stackelberg O, Orsini N, Wolk A, Bjorck M. Physical Activity, and Inactivity, and Risk of Abdominal Aortic Aneurysm. European Journal of Vascular and Endovascular Surgery Conference: The European Society for Vascular Surgery 32nd Annual Meeting 2018;(6 Supplement 2):December.

(5) Franks PJ, Edwards RJ, Greenhalgh RM, Powell JT. Risk factors for abdominal aortic aneurysms in smokers. Eur J Vasc Endovasc Surg 1996 May;11(4):487-92.

(6) Kelion AD, Banning AP, Gardner MA, Ormerod OJM. Exercise equilibrium radionuclide angiography predicts long-term cardiac prognosis in patients with abdominal aortic aneurysm being considered for surgery. Journal of Nuclear Cardiology 2000;7(3):249-54.

(7) Myers J, Mcelrath M, Jaffe A, Smith K, Fonda H, Vu A, Hill B, Dalman R. A randomized trial of exercise training in abdominal aortic aneurysm disease. Medicine and Science in Sports and Exercise 2014;46(1):2-9.

(8) Myers JN, White JJ, Narasimhan B, Dalman RL. Effects of exercise training in patients with abdominal aortic aneurysm: Preliminary results from a randomized trial. Journal of Cardiopulmonary Rehabilitation and Prevention 2010;30(6):374-83.

(9) Jamrozik K, Norman PE, Spencer CA, Parsons RW, Tuohy R, Lawrence-Brown MM, Dickinson JA. Screening for abdominal aortic aneurysm: lessons from a population-based study. Med J Aust 2000 Oct 2;173(7):345-50.

(10) Kent KC, Zwolak RM, Egorova NN, Riles TS, Manganaro A, Moskowitz AJ, Gelijns AC, Greco G. Analysis of risk factors for abdominal aortic aneurysm in a cohort of more than 3 million individuals. J Vasc Surg 2010 Sep;52(3):539-48.

(11) Golledge J, Hankey GJ, Yeap BB, Almeida OP, Flicker L, Norman PE. Reported high salt intake is associated with increased prevalence of abdominal aortic aneurysm and larger aortic diameter in older men. PLoS ONE 2014;9(7):e102578.

(12) Strachan DP. Predictors of death from aortic aneurysm among middle-aged men: the Whitehall study. Br J Surg 1991 Apr;78(4):401-4.

(13) Alcorn HG, Wolfson SK, Jr., Sutton-Tyrrell K, Kuller LH, O'Leary D. Risk factors for abdominal aortic aneurysms in older adults enrolled in The Cardiovascular Health Study. Arterioscler Thromb Vasc Biol 1996 Aug;16(8):963-70.

(14) Lee AJ, Fowkes FG, Carson MN, Leng GC, Allan PL. Smoking, atherosclerosis and risk of abdominal aortic aneurysm. Eur Heart J 1997 Apr;18(4):671-6.

(15) Watt HC, Law MR, Wald NJ, Craig WY, Ledue TB, Haddow JE. Serum triglyceride: a possible risk factor for ruptured abdominal aortic aneurysm. Int J Epidemiol 1998 Dec;27(6):949-52.

(16) Blanchard JF, Armenian HK, Friesen PP. Risk factors for abdominal aortic aneurysm: results of a case-control study. Am J Epidemiol 2000 Mar 15;151(6):575-83.

(17) Singh K, Bonaa KH, Jacobsen BK, Bjork L, Solberg S. Prevalence of and risk factors for abdominal aortic aneurysms in a population-based study : The Tromso Study. Am J Epidemiol 2001 Aug 1;154(3):236-44.

(18) Lederle FA, Johnson GR, Wilson SE. Abdominal aortic aneurysm in women. J Vasc Surg 2001 Jul;34(1):122-6.

(19) Rodin MB, Daviglus ML, Wong GC, Liu K, Garside DB, Greenland P, Stamler J. Middle age cardiovascular risk factors and abdominal aortic aneurysm in older age. Hypertension 2003 Jul;42(1):61-8.

(20) Cornuz J, Sidoti PC, Tevaearai H, Egger M. Risk factors for asymptomatic abdominal aortic aneurysm: systematic review and meta-analysis of population-based screening studies. Eur J Public Health 2004 Dec;14(4):343-9.

(21) Brady AR, Thompson SG, Fowkes FG, Greenhalgh RM, Powell JT. Abdominal aortic aneurysm expansion: risk factors and time intervals for surveillance. Circulation 2004 Jul 6;110(1):16-21.

(22) Wanhainen A, Bergqvist D, Boman K, Nilsson TK, Rutegard J, Bjorck M. Risk factors associated with abdominal aortic aneurysm: a population-based study with historical and current data. J Vasc Surg 2005 Mar;41(3):390-6.

(23) Iribarren C, Darbinian JA, Go AS, Fireman BH, Lee CD, Grey DP. Traditional and novel risk factors for clinically diagnosed abdominal aortic aneurysm: the Kaiser multiphasic health checkup cohort study. Ann Epidemiol 2007 Sep;17(9):669-78.

(24) Lederle FA, Larson JC, Margolis KL, Allison MA, Freiberg MS, Cochrane BB, Graettinger WF, Curb JD. Abdominal aortic aneurysm events in the women's health initiative: cohort study. BMJ 2008 Oct 14;337:a1724.

(25) Forsdahl SH, Singh K, Solberg S, Jacobsen BK. Risk factors for abdominal aortic aneurysms: a 7-year prospective study: the Tromso Study, 1994-2001. Circulation 2009 Apr 28;119(16):2202-8.

(26) Golledge J, van BF, Jamrozik K, McCann M, Norman PE. Association between serum lipoproteins and abdominal aortic aneurysm. Am J Cardiol 2010 May 15;105(10):1480-4.

(27) Solberg S, Forsdahl SH, Singh K, Jacobsen BK. Diameter of the infrarenal aorta as a risk factor for abdominal aortic aneurysm: the Tromso Study, 1994-2001. Eur J Vasc Endovasc Surg 2010 Mar;39(3):280-4.

(28) Sode BF, Nordestgaard BG, Gronbaek M, Dahl M. Tobacco smoking and aortic aneurysm: two population-based studies. Int J Cardiol 2013 Sep 1;167(5):2271-7.

(29) Smelser DT, Tromp G, Elmore JR, Kuivaniemi H, Franklin DP, Kirchner HL, Carey DJ. Population risk factor estimates for abdominal aortic aneurysm from electronic medical records: a case control study. BMC Cardiovasc Disord 2014 Dec 4;14:174.

(30) Howard DP, Banerjee A, Fairhead JF, Handa A, Silver LE, Rothwell PM. Age-specific incidence, risk factors and outcome of acute abdominal aortic aneurysms in a defined population. Br J Surg 2015 Jul;102(8):907-15.

(31) Jahangir E, Lipworth L, Edwards TL, Kabagambe EK, Mumma MT, Mensah GA, Fazio S, Blot WJ, Sampson UK. Smoking, sex, risk factors and abdominal aortic aneurysms: a prospective study of 18 782 persons aged above 65 years in the Southern Community Cohort Study. J Epidemiol Community Health 2015 May;69(5):481-8.

(32) Yuan H, Han X, Jiao D, Zhou P. A Case-Control Study of Risk Factors of Abdominal Aortic Aneurysm. Heart Surg Forum 2016 Oct 21;19(5):E224-E228.

(33) Han SA, Joh JH, Park HC. Risk Factors for Abdominal Aortic Aneurysm in the Korean Population. Ann Vasc Surg 2017 May;41:135-40.

(34) Wang L, Djousse L, Song Y, Akinkuolie AO, Matsumoto C, Manson JE, Gaziano JM, Sesso HD. Associations of Diabetes and Obesity with Risk of Abdominal Aortic Aneurysm in Men. Journal of Obesity 2017;3521649.

(35) Xiao J, Borne Y, Bao X, Persson M, Gottsater A, Acosta S, Engstrom G. Comparisons of Risk Factors for Abdominal Aortic Aneurysm and Coronary Heart Disease: A Prospective Cohort Study. Angiology 2020 Aug 7;3319720946976.

(36) Tang W, Yao L, Roetker NS, Alonso A, Lutsey PL, Steenson CC, Lederle FA, Hunter DW, Bengtson LG, Guan W, Missov E, Folsom AR. Lifetime Risk and Risk Factors for Abdominal Aortic Aneurysm in a 24-Year Prospective Study: The ARIC Study (Atherosclerosis Risk in Communities). Arterioscler Thromb Vasc Biol 2016 Dec;36(12):2468-77.

(37) Brown LC, Greenhalgh RM, Howell S, Powell JT, Thompson SG. Patient fitness and survival after abdominal aortic aneurysm repair in patients from the UK EVAR trials. British Journal of Surgery 2007;94(6):709-16.

(38) Fattahi N, Rosenblad A, Kragsterman B, Hultgren R. Risk factors in 50-year-old men predicting development of abdominal aortic aneurysm. Journal of Vascular Surgery 2020;72(4):1337-46.

Supplementary Table 2. Relative risks and 95% confidence intervals for the association between leisure-time physical activity and abdominal aortic aneurysm

| MET-hours/week | RRs (95% CIs) | RRs (95% CIs) sensitivity analysis excluding Hammond, 1969 study |
| --- | --- | --- |
| 0 | 1.00 | 1.00 |
| 5 | 0.92 (0.85-0.98) | 0.93 (0.87-1.00) |
| 10 | 0.85 (0.74-0.97) | 0.87 (0.76-1.00) |
| 15 | 0.79 (0.66-0.95) | 0.82 (0.68-1.00) |
| 20 | 0.75 (0.61-0.94) | 0.79 (0.63-0.99) |
| 25 | 0.73 (0.58-0.92) | 0.77 (0.60-0.98) |
| 30 | 0.72 (0.56-0.91) | 0.75 (0.58-0.97) |
| 35 | 0.71 (0.55-0.91) | 0.74 (0.57-0.97) |
| 40 | 0.71 (0.54-0.91) | 0.74 (0.56-0.97) |
| 45 | 0.71 (0.54-0.93) | 0.74 (0.56-0.98) |
| 50 | 0.71 (0.53-0.95) | 0.74 (0.54-1.00) |
| 55 | 0.71 (0.51-0.97) | 0.73 (0.53-1.02) |
| 59 | 0.71 (0.50-1.00) | 0.73 (0.51-1.05) |
| P_nonlinearity_ | 0.09 | 0.21 |

Supplementary Table 3. Study quality of included studies

| Author, publication year | Representative-ness | Selection of non-exposed cohort | Exposure ascertainment | Demonstration of outcome not present at start | Adjustment for one confounder | Adjustment for a second confounder | Assessment of outcome | Long enough follow-up | Loss to follow-up | Total |
| --- | --- | --- | --- | --- | --- | --- | --- | --- | --- | --- |
| Hammond, 1969 | 1 | 1 | 0 | 0 | 1 | 0 | 1 | 1 | 1 | 6 |
| Goldberg, 1995 | 0 | 1 | 0 | 1 | 1 | 1 | 1 | 1 | 0 | 6 |
| Tornwall, 2001 | 0 | 1 | 0 | 1 | 1 | 1 | 1 | 1 | 0 | 6 |
| Lindblad, 2005 | 1 | 1 | 0 | 0 | 1 | 1 | 1 | 1 | 0 | 6 |
| Wong, 2007 | 0 | 1 | 0 | 1 | 1 | 1 | 1 | 1 | 1 | 7 |
| Stackelberg, 2017 | 1 | 1 | 1 | 1 | 1 | 1 | 1 | 1 | 0 | 8 |
| Nordkvist, 2018 | 1 | 1 | 0 | 1 | 1 | 1 | 1 | 1 | 0 | 7 |
| Hamer, 2018 | 1 | 1 | 1 | 0 | 1 | 1 | 1 | 1 | 1 | 8 |
| Oyenuga, 2019 | 1 | 1 | 0 | 1 | 1 | 1 | 1 | 1 | 0 | 7 |

Supplementary Figure 1. Funnel plot of physical activity and abdominal aortic aneurysm

Supplementary Figure 2. Influence analysis of high vs. low analysis of physical activity and abdominal aortic aneurysm

------------------------------------------------------------------------------

Study omitted | e^coef. [95% Conf. Interval]

-------------------+----------------------------------------------------------

Oyenuga, 2019 | 0.68337065 0.51909798 0.8996287

Hamer, 2018 | 0.69211245 0.54958355 0.87160474

Nordkvist, 2018 | 0.74919134 0.60901839 0.92162681

Stackelberg, 2017 | 0.71406609 0.56302416 0.90562785

Wong, 2007 | 0.66088754 0.52680045 0.82910395

Lindblad, 2005 | 0.73940611 0.6022616 0.90778059

Tornwall, 2001 | 0.68576312 0.52847022 0.88987243

Goldberg, 1995 | 0.67036748 0.54158103 0.82977903

Hammond, 1969 | 0.71940523 0.5695349 0.90871334

-------------------+----------------------------------------------------------

Combined | 0.7009625 0.56406008 0.87109237

------------------------------------------------------------------------------

Supplementary Figure 3. Influence analysis of dose-response analysis of physical activity and abdominal aortic aneurysm

------------------------------------------------------------------------------

Study omitted | e^coef. [95% Conf. Interval]

-------------------+----------------------------------------------------------

Oyenuga, 2019 | 0.84221309 0.72227859 0.9820627

Hamer, 2018 | 0.82340854 0.71826053 0.94394952

Nordkvist, 2018 | 0.86198837 0.74566185 0.99646223

Stackelberg, 2017 | 0.85980678 0.75264496 0.98222637

Wong, 2007 | 0.81421697 0.72962403 0.90861768

Hammond, 1969 | 0.87031299 0.77956361 0.97162658

-------------------+----------------------------------------------------------

Combined | 0.8468857 0.75119363 0.95476766

------------------------------------------------------------------------------
